# Tanshinone IIA attenuates demyelination and promotes remyelination in* A. cantonensis*-infected BALB/c mice

**DOI:** 10.7150/ijbs.35266

**Published:** 2019-08-19

**Authors:** Ying Feng, Feng Feng, Cunjing Zheng, Zongpu Zhou, Meihua Jiang, Zhen Liu, Fukang Xie, Xi Sun, Zhongdao Wu

**Affiliations:** 1 Medical School of South China University of Technology, Guangzhou, China; 2 Parasitology Department of Zhongshan School of Medicine, Sun Yat-sen University, Guangzhou, China; 3 Key Laboratory of Tropical Disease Control (SYSU), Ministry of Education, Guangzhou, China; 4 The Department of Pharmacology and Toxicology, School of Pharmaceutical Sciences, Sun Yat-Sen University, Guangzhou, China; 5 Histology and Embryology Department of Zhongshan School of Medicine, Sun Yat-sen University, Guangzhou, China; 6 Anatomy Department of Zhongshan School of Medicine, Sun Yat-sen University, Guangzhou, China; 7 Guangzhou First People's Hospital, Guangzhou, China

**Keywords:** Tanshinone IIA, demyelination, remyelination, microglia polarization, A. *cantonensis*

## Abstract

Background: *Angiostrongylus cantonensis* infection can cause demyelination in the central nervous system, and there is no effective treatment. Methods: We used dexamethasone, Tanshinone IIA (TSIIA) and Cryptotanshinone(Two traditional Chinese medicine monomers) in combination with albendazole (AB, a standard anti-helminthic compound) to observe their therapeutic effect on demyelination in *A. cantonensis*-infected mice. Luxol fast blue staining and electron microscope of myelin sheath, Oligodendrocyte (OL) number and myelin basic protein (MBP) expression in brain was detected in above groups. Results: TSIIA+AB facilitated OL proliferation and significantly increased both myelin sheath thickness and the population of small-diameter axons. In addition, TSIIA treatment inhibited the expression of inflammation-related factors (interleukin [IL]-6, IL-1β, tumor necrosis factor [TNF]-α, inducible nitric oxide synthase [iNOS]) rather than inhibiting eosinophil infiltration in brain. TSIIA also decreased microglial activation and shifted their phenotype from M1 to M2. Conclusions: Taken together, these results provide evidence that TSIIA combined with AB may be an effective treatment for demyelination caused by *A. cantonensis* infection and other demyelinating diseases.

## Background

Angiostrongyliasis, a central nervous system (CNS) disease also called eosinophilia meningoencephalitis or eosinophilia myeloencephalitis, is caused by *Angiostrongylus cantonensis* infection by third-stage freshwater snail larvae (Larvae III). Eating raw or uncooked snail fish food that contains Larvae III is a common cause of infection. Larvae III mainly invade and infect the CNS and cause meningitis, spinal meningitis, encephalitis, and myelitis. The main clinical manifestation of angiostrongyliasis is eosinophilic meningitis. We and others have previously shown that *A. cantonensis* infection leads to obvious demyelination of CNS in BALB/c mice 1, 2. Previous studies revealed that anti-helminthic treatment alone or in combination with corticosteroids had no obvious effect on demyelination caused by *A. cantonensis* 2, 3, suggesting that simple antiparasitic or/and suppression of inflammation is not sufficient*.* It is therefore necessary to identify more efficient treatment strategies to prevent demyelination associated with angiostrongyliasis.

Myelin formed by oligodendrocytes (OLs) in the vertebrate CNS insulates axons to promote rapid, energy-efficient action potential propagation. Failed myelin repair after OL damage contributes to persistent demyelination in debilitating diseases such as multiple sclerosis (MS) and leukodystrophies 4. Some new drugs and stem cell therapy have opened new avenues for demyelination treatment, but current therapies are only partially effective or unsafe in the long term. Herbal therapies represent a promising therapeutic approach for demyelination due to their potent efficacy and superior safety profile 5.

Danshen is a Chinese herbal medicine that exerts antioxidant and anti-inflammatory actions in many experimental animal models. It is commonly used to treat cardiac and vascular disorders such as atherosclerosis and stroke 6, 7. Tanshinones are abietane diterpenes. Tanshinone IIA (TSIIA), cryptotanshinone, and tanshinone I are major bioactive constituents of Danshen, which consists of 0.29% TSIIA, 0.23% cryptotanshinone (CPT), 0.11% tanshinone I, and 0.054% 15, 16-dihydrotanshinone-I (Tian and Wu, 2013). Among the identified tanshinones, TSIIA and CPT have received the most attention, and they are the most abundant and well-studied constituents of Danshen. Over the past decade, TSIIA has been demonstrated to have potential protective effects against atherosclerosis, cardiac hypertrophy, cardiac fibrosis, diabetes, neurodegenerative diseases, and various cancers 8, 9. TSIIA reduces inflammation and demyelination in experimental autoimmune encephalitis (EAE) and optic neuritis caused by *A. cantonensis* 10, 11.

In this study, we applied CPT or TSIIA in combination with albendazole (AB) and measured demyelination caused by *A. cantonensis*. Our results indicate that TSIIA combined with AB can effectively attenuate demyelination and promote OL proliferation and remyelination, suggesting that this may be an effective method for treating demyelination caused by *A. cantonensis* infection and other demyelinating diseases.

## Materials and Methods

### Infection of mice with *A. cantonensis* larvae and subsequent treatment

BALB/c mice (20-40 g body weight, 6-8 weeks old) were purchased from the animal center laboratory at Sun Yat-sen University (Guangzhou, China) and infected with* A. cantonensis* larvae. The Institutional Animal Care and Use Committee approved all animal procedures. Larvae III (*L3*) of *A. cantonensis* were collected from giant African snails (*Achatina fulica*) via homogenization and digestion of minced snail tissue placed in a pepsin-HCL solution (pH 2.0, 500 IU pepsin/gram tissue) and incubated at 37 °C for 2 h. *L3* in the sediment were washed with phosphate-buffered saline (PBS) twice and counted under an anatomical microscope. The mice were randomly 12 divided into six groups (all n = 6): normal control, *A. cantonensis*-infected, AB, AB combined with dexamethasone, AB combined with CPT, and AB combined with TSII-A. All experiments were performed in triplicate. Besides the normal control group, all animals were infected with 30 *L3* by intragastric administration. At 14 d post-infection, the treatment groups were fed AB (20 mg/kg/d), AB combined with dexamethasone (0.5 mg/kg/d), AB combined with CPT (C_19_H_20_O_3_; MW: 296.33; HPLC: 90.5%; Hongsheng Biochem Ltd., Xi'an, China) with 50 mg/kg/d, or AB combined with TSIIA (C_19_H_18_O_3_; MW: 294.36; HPLC:
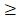
98%; Hongsheng Biochem Ltd.) with 50 mg/kg/d, respectively. The normal and infected groups were fed the same amount of vehicle (dimethyl sulfoxide, DMSO). Drugs or DMSO were administered via gastric infusion to mice once daily for 7 d. We also established a sole TSII-A treatment group with *A. cantonensis* to detect the effect of TSII-A on killing *A. cantonensis* (same method as above).

### Neurobehavioral scores

At 21 d post-infection, neurological assessments for motor and sensory function were performed by staff blinded to group assignments. Motor function encompassed freedom of movement, limb symmetry, climbing, and balance. Sensory function included proprioception; tentacles reaction; and visual, olfactory, and pain responses. Every test was graded on a scale from 0 to 3, and the total for all items was 27 (for details on behavioral scoring, please refer to 13. The neurological function score of each mouse was the sum of the scores on all of the above tests.

### *A. cantonensis* larvae IV (*L4*) collection and quantification

The mice in *A. cantonensis*-infected and TSII-A treatment groups were sacrificed by chloral hydrate asphyxiation and cervical dislocation at 21 d post-infection. Whole brains were removed and placed in PBS to promote the escape of *A. cantonensis* from the brain. The number of *A. cantonensis* in the solution for each mouse was counted to calculate the insect detection rate (number of *A. cantonensis/*30).

### Histopathological observation

Mice were sacrificed by chloral hydrate asphyxiation and cervical dislocation at 21 d post-infection. The brains were quickly dissected and fixed in 4% paraformaldehyde overnight. Paraffin-embedded sections were then prepared. Hematoxylin and eosin (HE) staining 14 was performed, and the meninges and cerebral cortex were observed and imaged under microscopy (Olympus, Tokyo, Japan).

### Luxol fast blue (LFB)

The brain samples were dehydrated in a gradient alcohol series, cleared in xylene, and embedded in paraffin. Sections (5-μm thick) were cut on an RM 2035 microtome (Reichert S, Leica, Wetzlar, Germany). Myelin was detected using Luxol fast blue (LFB) staining 15 (Solvent Blue 38; Sigma-Aldrich, St. Louis, MO). The sections were observed under an inverted microscope equipped with a camera (DM 2500B, Leica).

### Cell count

Blood samples were collected in tubes with 100 IU/mL heparin sodium, then detected with a hematology analyzer for animals (Sysmex, Kobe, Japan). Numbers of eosinophils in the blood were recorded and compared among the different groups.

### Transmission electron microscopy (TEM)

After anesthesia, the animals were sacrificed by transcardial perfusion with 4% paraformaldehyde. The eyes were quickly removed and post-fixed overnight in 2.5% glutaraldehyde. Next, the optic nerves were post-fixed in a solution containing 1% osmium tetroxide (Sigma-Aldrich). Then, they were dehydrated in an acetone series and embedded in SPIN-PON resin. Resin polymerization was performed at 60 °C for 3 days. Semi-thin sections (0.5-μm thickness) were placed on glass slides and stained with toluidine blue. Finally, demyelination detection 16 was done by using a 300KV transmission electronic microscope (FEI, Hillsboro, OR), and three image fields were taken from each specimen of every group. These images were used to measure the numbers and diameters of axons in Image-Pro Plus 6.0 software (Media Cybernetics, Rockville, MD).

### Immunofluorescence

After fixing with 4% paraformaldehyde, the mouse brain and optic nerve sections were cut at 15-μm thickness at -20 °C and mounted on glass slides. Then, sections were blocked with 3% bovine serum albumin (BSA) at room temperature for 1 h before incubation with rabbit anti-mouse CC1 (OP80, Merck, Kenilworth, NJ), mouse anti-O4 (O7139, Sigma), rabbit anti-ki67 (ab833, Abcam, Cambridge, UK), rabbit-anti-arg-1 (ab91279, Abcam), mouse anti-Iba-1 (SAB2702364, Sigma), and rabbit anti-inducible nitric oxide synthase (iNOS; ab15323, Abcam) monoclonal antibodies in 1% BSA at 4 °C overnight. Sections were washed three times in PBS, incubated with tetramethylrhodamine (TRITC)-labeled and fluorescein isothiocyanate (FITC)-labeled secondary antibody (Abcam) diluted 1:500 in 1% BSA at 37 °C for 1 h, and washed again in PBS. Then, DAPI (1:1000 dilution, 4083S Beyotime Biotechnology, Haimen, China) was used to stain the nuclei for 5 min. Specimens incubated without a primary antibody was used as negative controls. Slides were observed under a confocal microscope.

### RNA isolation and real-time quantitative polymerase chain reaction (PCR)

Total RNA was extracted from cells with TRIzol reagent based on previous studies 17. For cDNA synthesis, RNA was reverse transcribed with a PrimeScript RT reagent kit (TaKaRa, Kusatsu, Japan). The expression levels of the genes encoding interleukin (IL)-1β, iNOS, tumor necrosis factor (TNF)-α, IL-6, IL-5, IL-13, CD86, CD206, Ym-1, CD11b, and Arg-1 were measured by real-time PCR with SYBR Premix *Ex Taq* kit (TaKaRa). cDNA amplification was performed on an ABI Prism 7900 HT cycler (Applied Biosystems, Foster City, CA). mRNA levels were measured by using the specific primer sets listed in Table 1.

### Western blotting

The brain tissues were washed twice with cold PBS and lysed in extraction buffer (20 mM HEPES [pH 7.4], 2 mM EDTA, 50 mM glycerophosphate, 1 mM dithiothreitol, 1 mM Na_3_VO_4_, 1% Triton X-100, and 10% glycerol) on ice. The lysates were centrifuged at 12,000 rpm for 15 min. Supernatants were collected, and protein concentrations were determined by bicinchoninic (BCA) Protein Assay (Bio-Rad, Hercules, CA). Proteins were boiled in sample buffer, separated in 12% sodium dodecyl sulfate-polyacrylamide gels by electrophoresis (50 μg/lane), and electroblotted onto nitrocellulose membranes (Pall Corporation, Ann Arbor, MI). Transferred blots were incubated sequentially with blocking agent (5% non-fat milk in Tris-buffered saline), anti-iNOS antibody (1:125 dilution, ab15323, Abcam), anti-Arg-1 antibody (1:500 dilution, ab91279, Abcam), and secondary antibody blots prior to development with enhanced chemifluorescence detection kits and exposure Hyperfilm (Fuji, Tokyo, Japan) according to the manufacturer's directions. The same blots were subsequently stripped and reblotted with antibodies that recognized β-actin (Sigma) as a reference to verify equal amounts of the protein among samples. Graphs of blots were obtained in the linear range of detection and were quantified for the level of specific induction with the Scion Image System.

### Statistics

One-way analyses of variance (ANOVAs) were used to compare neurobehavioral scores, myelin sheath number, real-time PCR data, and western blotting data among groups. Statistics were performed using IBM SPSS statistics 19 (IBM Corp, Armonk, NY). For all tests, P < 0.05 was considered statistically significant.

## Results

### TSIIA combined with AB improved neurobehavioral scores

**Figure 2:**
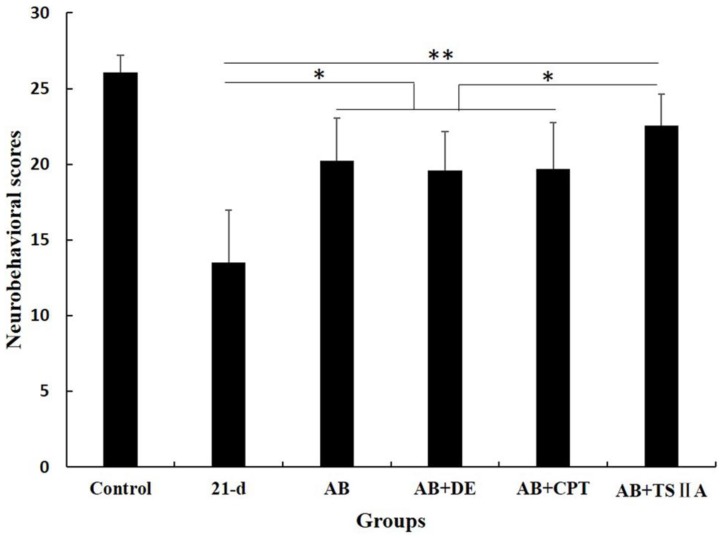
** Neurological function scores** of A. cantonensis-infected mice after treatment with albendazole (AB) alone, albendazole combined with dexamethasone (De), albendazole combined with CPT, or albendazole combined with TSIIA for 7 d. *P < 0.05, **P < 0.01. n = 6 per group and repeated the experiment three times.

To observe the effects of different therapy methods on angiostrongyliasis, the neurobehavioral scores of infected mice in each group were evaluated. All treatment methods led to some improvement compared with untreated 21-d *A. cantonensis-infected* mice, but TSIIA combined with AB was more effective at improving neurological behaviors than AB alone, dexamethasone combined with AB, and CPT combined with AB. The neurobehavioral scores in this treatment group recovered to normal (Figure 2).

### TSIIA combined with AB protected OLs from damage and attenuated demyelination significantly more than other treatments

**Figure 3:**
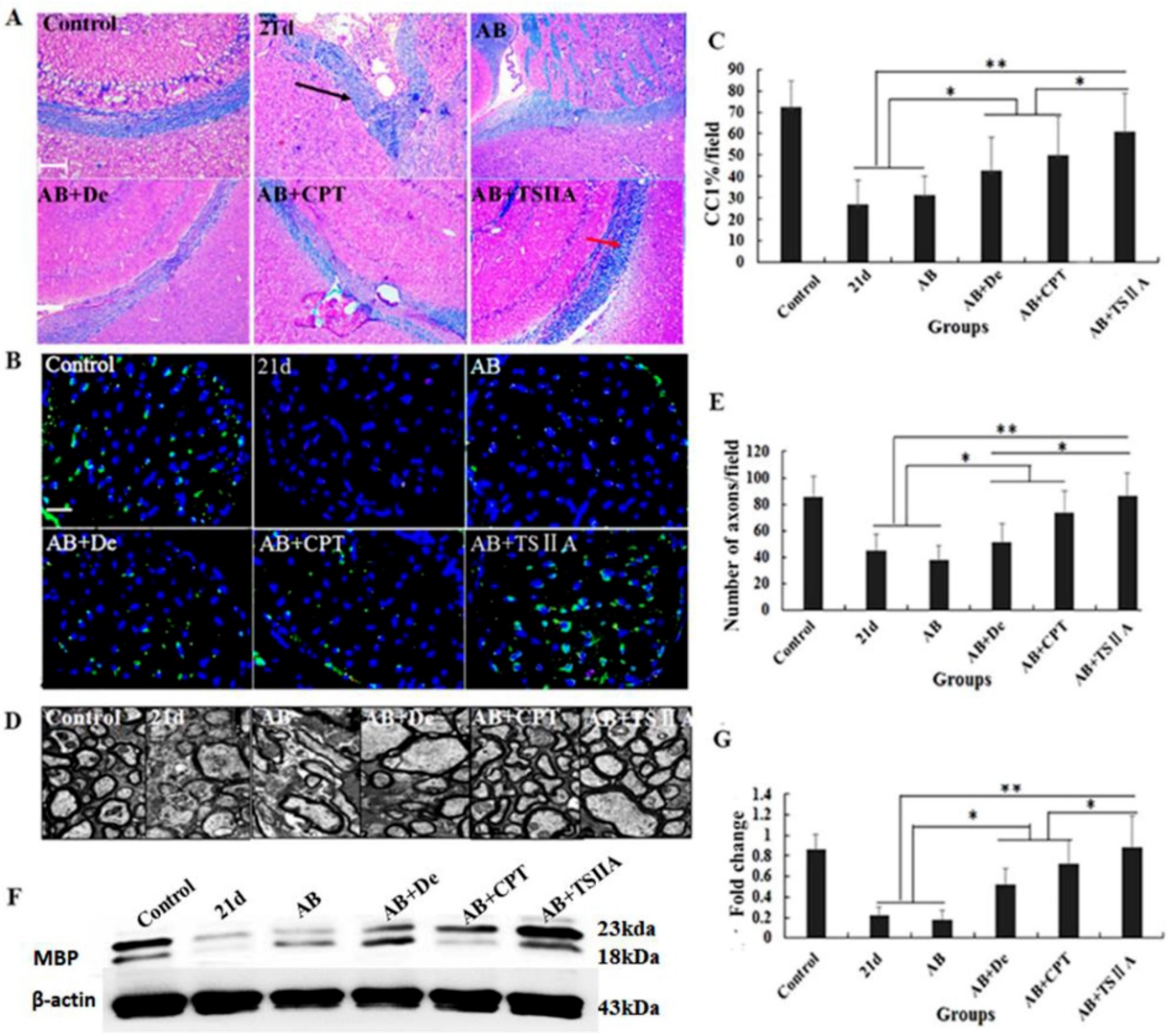
** TSIIA combined with albendazole reduced demyelination more than other treatments in *A. cantonensis*-infected mice. (A)** Luxol fast blue (LFB) staining revealed corpus callosum demyelination of mice in the control group, 21-d A. cantonensis infection group, AB treatment group, AB+De treatment group, AB+CPT treatment group, and AB+TSIIA treatment group. Black arrow indicates decreased myelin sheath after 21-d A. cantonensis infection, yellow arrows indicate deeper-staining myelin sheathes in 21-d *A. cantonensis* infected-mice treated with AB+TSⅡA. **(B)** CC1-positive cells present in the optic nerve were immunolabeled with CC1 antibodies (Green). CC1-positive cells decreased after 21-d *A. cantonensis* infection. AB, AB+De, and AB+CPT treatment did not obviously increase CC1-positive cells compared to AB+TSIIA treatment. **(C)** The percentage of CC1-positive cells (number of positive cells/DAPI-positive nuclei in one image) in the optic nerve in different groups. There were significantly fewer CC1-positive cells in the 21-d A. cantonensis infection and AB treatment groups compared to control. **(D)** TEM images of the optic nerve in different treatment groups. In the group infected with *A. cantonensis* for 21 d, optic nerve fibers exhibited obvious demyelination.** (E)** Quantification of optic nerve axons in the different treatment groups. **(F), (G)** Western blotting of MBP expression in the brain tissue of mice in the different treatment groups. Numerical results are presented as mean ± SEM. Student's t tests were used; n = 3 animals per group, experiments performed in triplicate, and at least three fields were analyzed per section in at least two non-adjacent sections per animal. Scale bars in A and C are 50 μm and 1 μm, respectively. *P < 0.05.

The above results showed that TSIIA treatment was an effective method to improve neurological behaviors and protect nerves from demyelination in the setting of angiostrongyliasis. To study the effect of different treatment methods on *A. cantonensis*-induced demyelination, we examined LFB staining of the brain and CC1 (mature OL marker) expression in the optic nerve. TEM was performed to visualize the optic nerve and quantify the numbers of myelin sheaths. Our results demonstrated that in the 21-d infection group, LFB staining was narrow, and vacuoles were obvious in high-power fields. This was also the case in the AB alone and AB combined with dexamethasone treatment groups. In the AB combined with CPT treatment group, LFB staining was deeper than in the 21-d infection group, but vacuoles were still obvious. In the TSIIA combined with AB treatment group, there were no differences from the normal control in the low- and high-power fields (Figure 3A). Immunofluorescence results showed that a decrease in CC1-positive cells in the 21-d infection and AB alone treatment group compared to the normal control. AB alone or in combination with dexamethasone did not increase the number of CC1-positive cells. However, the percentages of CC1-positive cells in the AB-CPT and AB-TSIIA combination groups were significantly higher than in the 21-d infection group. The effect in the AB-TSIIA combined treatment group was more obvious than in the other groups and was not significantly different to the normal control group with regard to the percentage of CC1-positive cells (Figure 3B, 3C). This indicated that *A. cantonensis* can induce OL damage and that TSIIA may have a protective effect. AB alone was not able to rescue these changes. The TEM analysis of nerve fibers also showed *A. cantonensis* infection led to demyelination and axon loss. AB with or without dexamethasone also had no obvious effect on demyelination. In contrast, AB combined with CPT or TSIIA appeared to have a good curative effect on the demyelination and axon loss caused by *A. cantonensis*, but TSIIA had a stronger impact (Figure 3D, 3E). We also observed a definite reduction in MBP protein expression at 21 d after *A. cantonensis* infection; AB with or without dexamethasone was unable to ameliorate the reduction, but AB combined with CPT or TSIIA obviously increased MBP expression (Figure 3F, 3G). These results demonstrate that AB combined with TSIIA attenuates demyelination induced by *A. cantonensis* infection.

### TSIIA combined with AB effectively promoted remyelination

**Figure 4:**
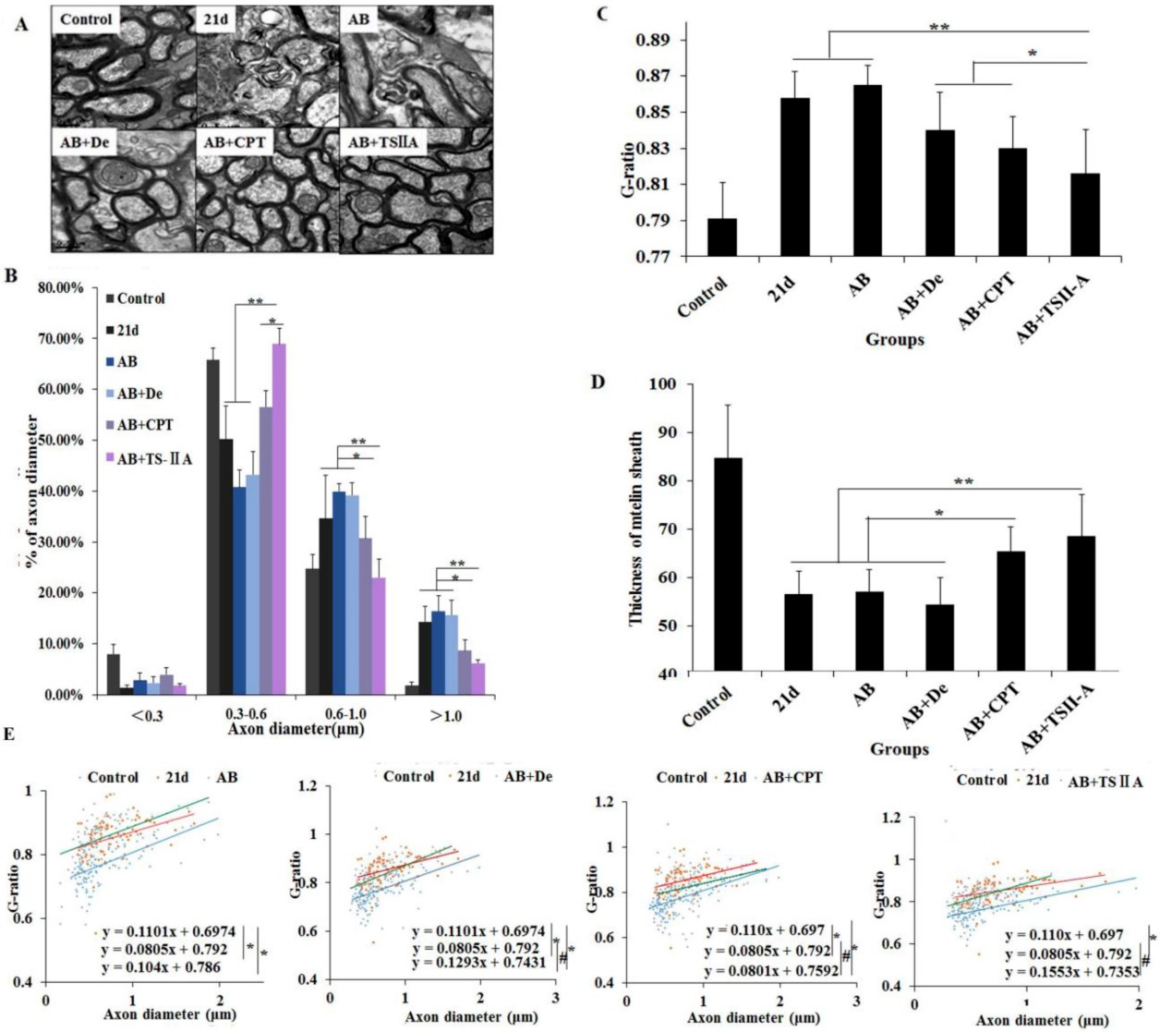
**TSIIA combined with albendazole promoted remyelination in *A. cantonensis*-infected mice. (A)** TEM images of the optic nerve in different treatment groups. The data of B, C, and D were derived from the images in A. Two-way ANOVA with Bonferroni's multiple comparisons test was used. **(B)** Percentages of myelinated optic nerve axons with various diameters in the different treatment groups. **(C)** The g-ratios of myelinated axons in the optic nerve for different groups. **(D)** Myelin sheath thicknesses of the optic nerve in the different treatment groups.** (E)** The g-ratio was plotted against the axon diameter. The best-fit lines obtained by linear regression differed significantly between the control and other groups, except for the AB+TSIIA treatment group. Moreover, the best-fit lines of the 21-d A. cantonensis infection and other treatment groups, except the AB group, were also significantly different. n = 3 animals per genotype, experiments performed in triplicate. At least 200 axons were analyzed per animal. * P < 0.05, **P < 0.01.

We further evaluate the effects of different treatments on remyelination. At 21 d after *A. cantonensis* infection, there was an evident increase in large-size axons (>1.0 μm) and a decrease in small-size axons (<0.5 μm) compared with the normal control group, which indicated that *A. cantonensis* infection can cause optic nerve fiber swelling and damage. After treatment, the small-size axons were more obviously increased in the AB+TSIIA group compared to the other treatment groups (Figure 4B), suggesting there were more remyelinated axons in this group 18, 19. In contrast, the other treatment groups had no marked change in axon diameter after *A. cantonensis* infection. At the same time, we quantified the g-ratio (axon circumference to myelin circumference), which provides a measure of the myelin thickness that complements axon morphology (diameter and density) in the assessment of demyelination in diseases 18, 20. The results confirmed that the fully compacted myelin sheaths formed in the TSIIA combined with AB treatment group were thicker than those in other treatment groups. Moreover, the g-ratio of the AB combined with TSIIA treatment group was not significantly different from that of the normal group, whereas the other groups were significantly different from the control (Figure 4C). In addition, myelin sheath thickness in the AB combined with TSIIA and AB combined with CPT groups recovered to normal levels, but the other groups were still obviously different from the control group (Figure 4D). These observations suggest that both AB combined with TSIIA and AB combined with CPT promoted optic nerve remyelination, but TSIIA was more effective. When this ratio was plotted against the axon diameter, lower values were seen for all axon diameters in the optic nerve, and the equations for the best-fit lines obtained by linear regression for the AB combined with TSIIA treatment group datasets differed significantly from that for the 21-d infection group but there was no difference with the control group. The other treatment groups were different from the 21-d infection and control groups (Figure 4E), which indicated axons that are myelinated in the TSIIA combined with AB treatment group have thicker sheaths than axons of similar diameters in other treatment groups and 21d *A. cantonensis*-infected mice. Collectively, these results suggest that AB combined with TSIIA had a better effect on remyelination than other treatments.

### TSIIA combined with AB contributed to OL proliferation and differentiation

**Figure 5:**
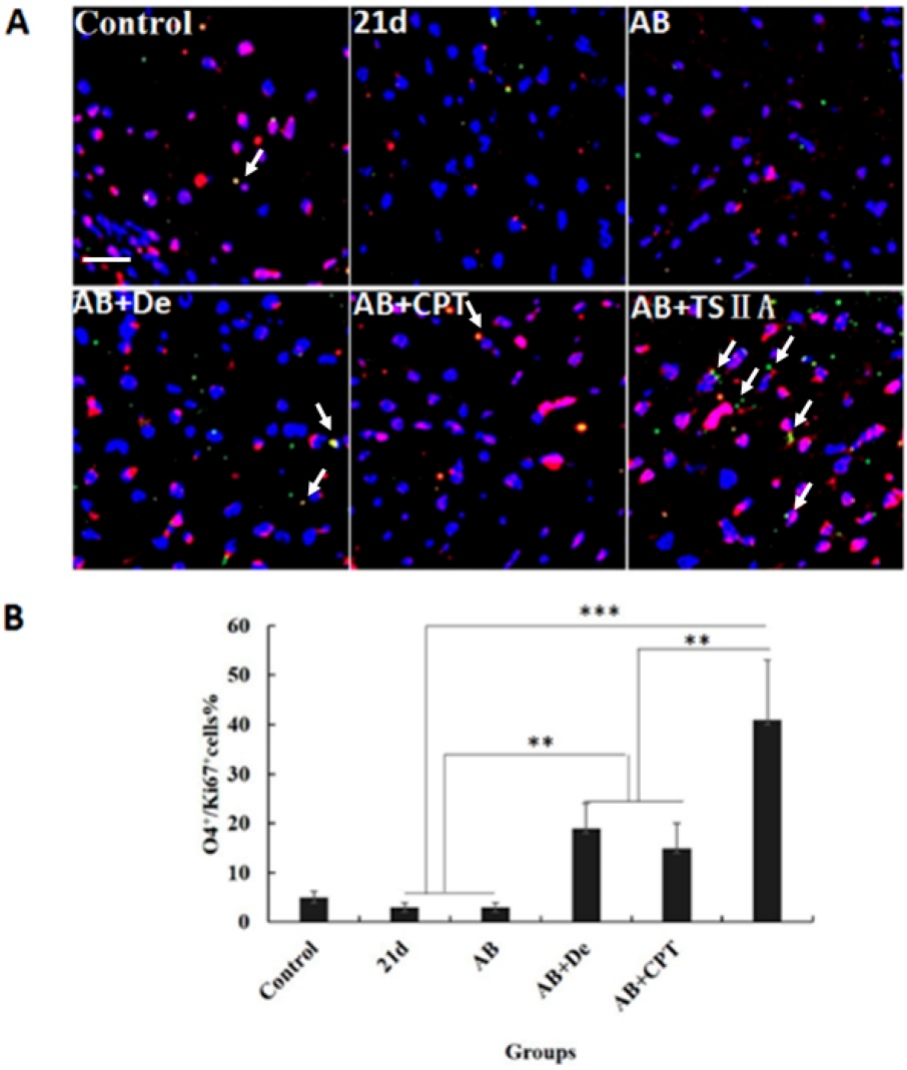
** TSIIA combined with albendazole contributed to OL proliferation and differentiation in *A. cantonensis*-infected mice. (A)** O4 and Ki67 double-positive cells present in the optic nerve were immunolabeled with antibodies for O4 (Green) and ki67 (red). White arrows indicate double-positive cells.** (B)** The percentage of O4-Ki67 double-positive cells present in the optic nerves of different groups. Numerical results are presented as the mean ± SEM. Student's t tests were used; n = 3 animals per group, experiments performed in triplicate, and at least three fields were analyzed per section in at least two non-adjacent sections per animal. Scale bar: 50 μm. * P < 0.05, **P < 0.01, ***P < 0.001.

The results above revealed that TSIIA combined with AB more effectively protected OLs and prevented demyelination than the other treatments. However, it is unknown whether these therapy methods also promote remyelination. This process of demyelination protection is initiated by activation of OL progenitor cells, so we investigated OL proliferation and differentiation. We examined O4 (OL progenitor marker) and Ki67 expression in optic neuritis and found that the percentage of O4 and Ki67 double-positive cells increased slightly in the AB combined with dexamethasone treatment group compared with the normal control, 21d-*A. cantonensis* infection, and AB alone treatment groups. Notably, the AB combined with TSIIA group had the most O4-Ki67 double-positive cells compared to the other treatment groups. These findings indicate that both dexamethasone and TSIIA can protect OLs from death and contribute to OL proliferation, but TSIIA may have a stronger effect on remyelination than dexamethasone (Figure 5A, 5B).

### TSIIA-induced remyelination was not achieved by inhibiting eosinophil infiltration and reducing *A. cantonensis* but by restraining inflammatory cytokine expression in the brain

**Figure 6:**
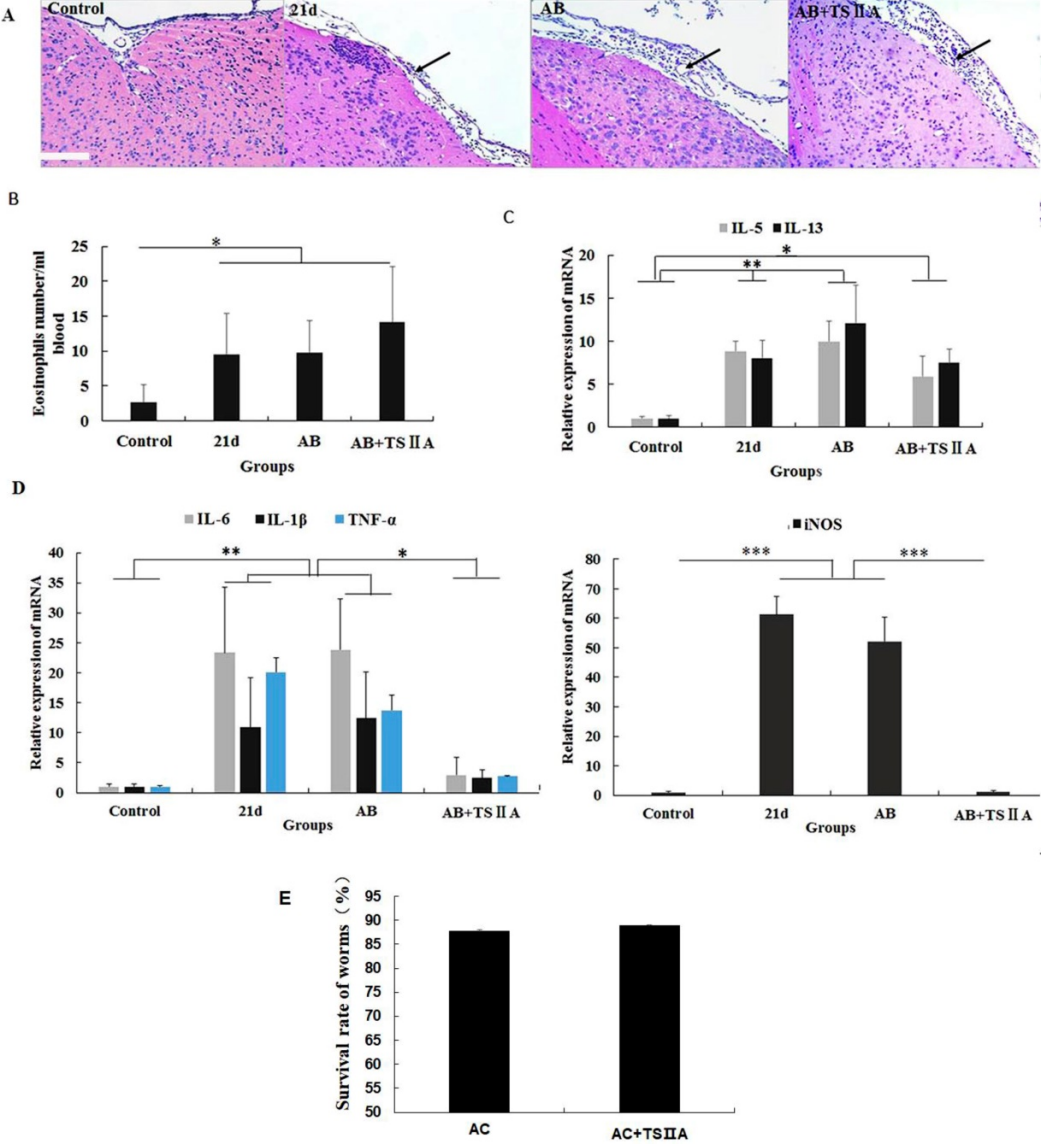
** The remyelination effect of TSIIA was not achieved by inhibiting eosinophil infiltration into the brains of *A. cantonensis*-infected mice. (A)** HE staining of the brain. In the 21-d* A. cantonensis* infection group, there was obvious inflammatory cell infiltration of the meninges and cerebral cortex. In the AB and AB+TSIIA treatment groups, inflammation was still observed in the meninges (black arrows). **(B)** The number of eosinophils in the blood. **(C)** mRNA expression of IL-5 and IL-13. **(D)** mRNA expression of iNOS, IL-1β, TNF-α, and IL-6. Numerical results are presented as mean ± SEM. Student's t tests were used; n = 3 animals per group, experiments performed in triplicate, and at least three fields were analyzed per section in at least two non-adjacent sections per animal. **(E)**Survival rate of worms in 21-d* A. cantonensis* infection group and TSIIA treatment groups. There were no significant differences between the two groups. Scale bar: 50 μm. * P < 0.05, **P < 0.01, ***P < 0.001.

To explore how TSIIA can inhibit demyelination and promote remyelination in *A. cantonensis*-infected mice, we performed HE staining of the brain, cell counting with a hematology analyzer, and real-time PCR to detect inflammatory infiltration of the brain, the number of eosinophils in blood, and mRNA levels of eosinophil-related cytokines (IL-5 and IL-13) and inflammatory factors (iNOS, TNF-α, IL-6, and IL-1β). To examine the effect of TSIIA on *A. cantonensis*, we measured survival rate of worms in *A. cantonensis*-infected and only TSIIA treatment groups. The experimental groups were normal control, 21-d *A. cantonensis*-infected group, AB treatment group, and AB combined with TSIIA treatment. *A. cantonensis* infection caused obvious eosinophil infiltration of the cerebral meninges, and eosinophils increased in the blood of 21-d infection group. AB alone or in combination with TSIIA had no apparent effect on eosinophil infiltration or quantity (Figure 6A, 6B). These results indicate that the alleviating effect of TSIIA on demyelination is not achieved by inhibiting eosinophil infiltration into the brain. Moreover, real-time PCR showed increased expression of the eosinophil-related cytokines IL-5 and IL-13 upon *A. cantonensis* infection, but there was no apparent difference before and after treatment with AB alone or in combination with TSIIA (Figure 6C). However, the increased expression levels of inflammation-related factors iNOS, IL-6, IL-1β and TNF-α upon *A. cantonensis* infection were not affected by AB treatment but decreased obviously after AB combined with TSIIA treatment. Moreover, iNOS expression was down-regulated much more apparently than other cytokines and even reached normal levels (Figure 5D). Finally, the survival rate of worms in the *A. cantonensis*-infected and only TSIIA treatment groups was not significantly different, suggesting that TSIIA did not kill* A. cantonensis*. These results suggest that the demyelination and remyelination effects of TSIIA are achieved via effects on inflammation-related signaling pathways rather than by inhibiting eosinophil infiltration into the brain or reducing the number of *A. cantonensis.*

### Polarization of microglia from M1 to M2 may underlie the remyelination effect of TSIIA

**Figure 7:**
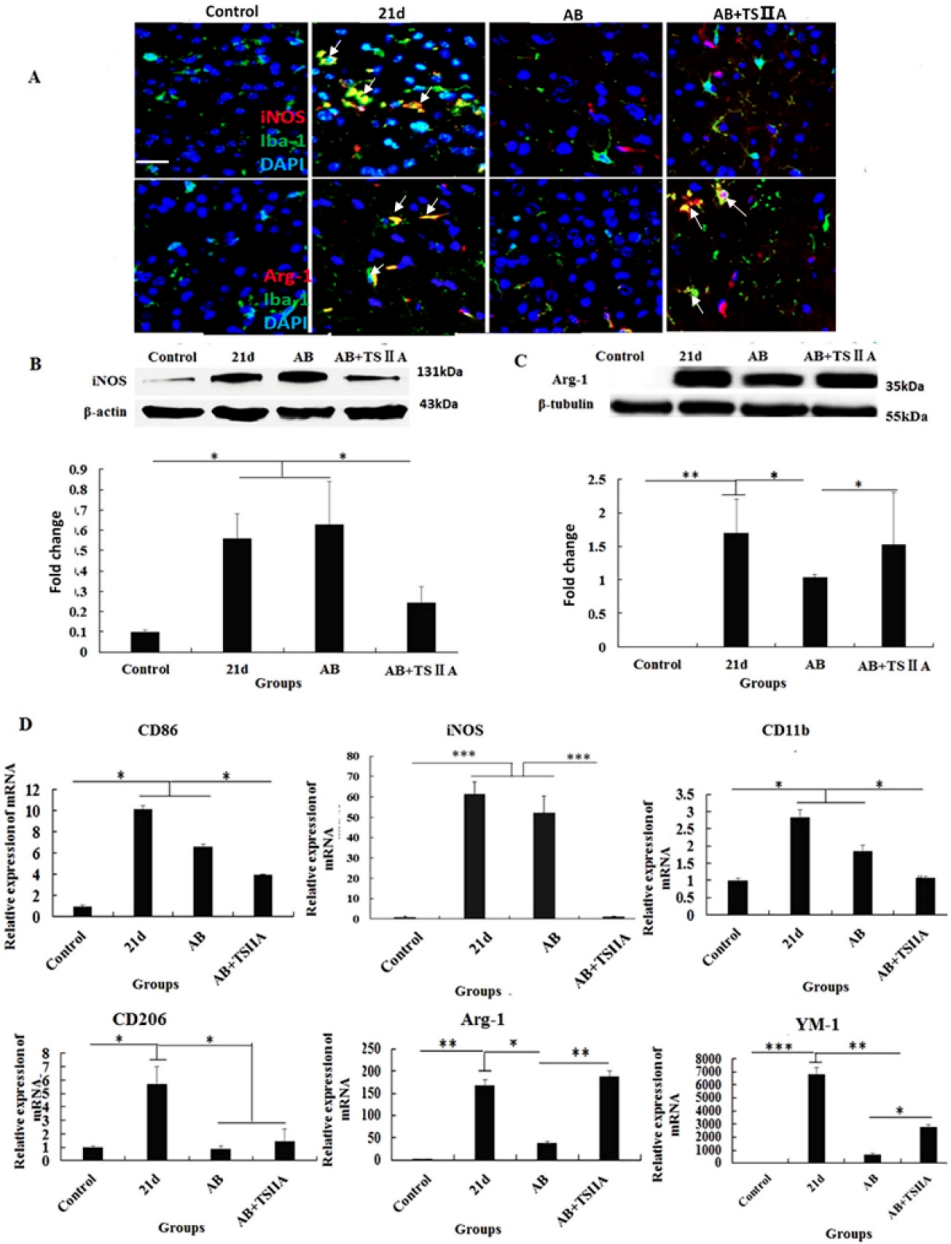
** The polarization of microglia from M1 to M2 may be the key reason underlying TSIIA-induced remyelination. (A)** iNOS/Iba-1- and Arg-1/Iba-1-positive cells in the brain tissue of mice were immunolabeled with antibodies against iNOS/Arg-1 (red) and Iba-1 (green). White arrows indicate iNOS/Iba-1- or Arg-1/Iba-1-positive cells. **(B), (C)** Western blotting of iNOS and Arg-1 in mouse brain tissue. **(D)** mRNA expression of M1 microglial markers (CD86, iNOS, CD11b) and M2 markers (CD206, Arg-1, YM1). Numerical results are presented as mean ± SEM. Student's t tests were used; n = 3 animals per group, experiments performed in triplicate, and at least three fields were analyzed per section in at least two non-adjacent sections per animal. * P < 0.05, **P < 0.01, ***P < 0.001.

The above results showed that inhibition of the aforementioned pro-inflammatory factors may take part in attenuating demyelination and promoting remyelination. M1 microglial cells are known to be pro-inflammatory, while M2 cells are anti-inflammatory. Furthermore, M2 microglia cells have regenerative capacity and are required for OL differentiation. iNOS and Arg-1 are considered markers of M1 and M2 microglia, respectively. However, whether M1 and M2 microglial cells take part in the remyelination function of TSIIA is still unknown. To resolve this question, immunofluorescence, western blotting, and real-time PCR were performed to examine whether M1 microglia polarized to M2 when *A. cantonensis*-infected mice were treated with both AB and TSIIA. After 21 d of *A. cantonensis* infection, Iba-1 (microglial marker) -positive cells also expressed iNOS. When treated with AB combined with TSIIA, iNOS expression decreased and Arg-1 expression increased in Iba-1 positive cells (Figure 7A). Western blotting confirmed that iNOS expression decreased and Arg-1 expression increased after combined AB and TSIIA treatment. Real-time PCR revealed that expression levels of M1 microglial cell markers (CD86, iNOS, CD11b) and M2 microglial cell markers (CD206, Arg-1, YM1) were elevated in the 21 d A. *cantonensis-*infected group and declined after AB treatment. On the other hand, markers of M1 microglial cells decreased further and those of M2 microglial cells increased significantly after TSIIA treatment (Figure 7B) compared with the AB-alone treatment group. Our results indicate that TSIIA may alleviate demyelination and promote remyelination in *A. cantonensis*-infected mice by polarizing microglia from M1 to M2.

## Discussion

A previous study reported that *A. cantonensis* can invade the CNS and cause demyelination followed by eosinophilic encephalitis 1, but the mechanisms are still unknown. Some surveys showed that anti-inflammation or anti-helminthic treatment had no obvious effect on demyelination caused by *A. cantonensis*. We therefore investigated the impact of TSIIA combined with AB 2, 3 on demyelination caused by *A. cantonensis*. Previous studies showed that TSIIA can protect neurons from damage, including ganglion cells, dopaminergic neurons, hippocampal neurons, and cortical neurons 21-24. However, its role in OL protection and remyelination has never been explored.

In this study, TSIIA combined with AB improved motor and sensory function as demonstrated by the neurobehavioral scores of *A. cantonensis-*infected mice compared with those in the other treatment groups. This may be caused by the attenuation of demyelination and OL damage since demyelination has been proposed as the predominant reason for axonal and neuronal degeneration that lead to abnormal neurobehavior 25. Our findings support the hypothesis that TSIIA combined with AB was more effective than other treatments on protecting demyelination in *A. cantonensis-*infected mice. LFB staining, OL and axon quantification, and MBP expression in the brain indicated that demyelination caused by *A. cantonensis* was significantly attenuated significantly by TSIIA and AB combined treatment*.* The present study therefore provides evidence that TSIIA mitigates demyelination, which is in agreement with previous reports.

In some diseases, remyelination occurs as a spontaneous regenerative process following demyelination 26. Previous studies have shown that remyelination is mediated by OL precursor cells (OPCs) that are widely distributed throughout the adult CNS 27. Remarkably, we found that TSIIA can promote remyelination in response to demyelination induced by *A. cantonensis.* We quantified g-ratios, axon numbers, myelin sheath thickness, and axon diameters, which are reliable indicators of remyelination. It is worth mentioning that TEM was used to evaluate de- and remyelination in the optic nerve because it is also affected by *A. cantonensis* impairment. The normal optic nerve is composed entirely of myelinated nerve fibers, making it ideal for observing myelination without interference from unmyelinated nerve fibers. In addition, OLs constitute approximately two-thirds of the interstitial cells behind the lamina cribrosa 28, so we observed OL proliferation and differentiation in the optic nerve as a measure of remyelination. As expected, the results of the above three remyelination indicators showed that TSIIA treatment can promote remyelination. Moreover, O4 and Ki67 immunofluorescence in brain sections confirmed that both OPC quantity and the cell proliferation ability of the optic nerve were obviously higher in the TSIIA combined with AB treatment group than in the other groups. This supports our proposal that TSIIA may facilitate OL proliferation and differentiation to promote new myelin sheath formation and remyelination. Based on these results, TSIIA may mainly function by promoting remyelination, but its role in myelin protection cannot be excluded. In any case, TSIIA can increase myelin density. To our knowledge, this is the first study to describe the function of TSIIA on OL protection and remyelination, but the mechanism requires further investigation.

Previous studies reported that eosinophilic infiltration of the brain was an important feature of *A. cantonensis-*infected mice 29, 30 and may be a possible reason for neurological damage 31. Based on this, we hypothesize that decreased demyelination may be due to inhibition of eosinophilic infiltration in CNS. We measured eosinophil numbers in the blood and eosinophil-related inflammatory factors in the brains of mice. To exclude the possibility that TSIIA attenuated eosinophilic inflammation by reducing the number of parasites*,* we also quantified the survival rate of worms*.* Unlike dexamethasone combined with AB, TSIIA combined with AB was unable to alleviate eosinophilic inflammation of the meninges or decrease the number of eosinophils in blood. Combined treatment did not affect mRNA levels of the eosinophil-related cytokines IL-5 and IL-13. Furthermore, TSIIA had no influence on the number of *A. cantonensis*, which further indicated that the beneficial effects of TSIIA on myelination were not facilitated through inhibition of eosinophil infiltration.

Recent years have witnessed an explosion of studies identifying factors for efficient remyelination, both extrinsic (also described as environmental or non-cell autonomous, such as inflammation) and intrinsic (cell autonomous) 32. The neuroinflammatory response modulates both the pathological changes of demyelination and axonal injury and remyelination and repair 33. It is becoming apparent that inflammatory response activation is essential component of tissue regeneration and remyelination 34, 35. When an eosinophilic mechanism for TSIIA effects on demyelination and remyelination was excluded, we speculated that inflammation may be the key reason for TSIIA efficacy following *A. cantonensis* infection. To clarify the mechanism, we measured the expression levels of the inflammation-related cytokines iNOS, IL-1β, TNF-α, and IL-6. The mRNA levels of all four cytokines increased after 21 d of *A. cantonensis* infection and were obviously decreased in mice treated with both TSIIA and AB. This result indicates that TSIIA may reduce pro-inflammatory cytokine levels to alleviate demyelination and promote remyelination.

Emerging evidence indicates that macrophages and microglia also play critical roles in modulating demyelination and remyelination through their antigen-presenting ability and production of cytokines, chemokines, and growth factors 36-38. In the CNS, microglias have two distinct functional phenotypes: pro-inflammatory (M1) and anti-inflammatory (M2). The M1 classically activated' phenotype is associated with enhanced antigen-presentation properties and secretion of pro-inflammatory cytokines and reactive oxygen and nitrogen species, whereas M2 microglia secrete anti-inflammatory cytokines and growth factors to exert neuroprotective effects 17, 39, 40. In our study, immunofluorescence and protein detection analyses showed increased M1 and M2 marker levels after 21 d of *A. cantonensis* infection. These were remarkably reduced after treatment with AB, which indicated that *A. cantonensis* infection can activate M1 and M2 microglia and that AB treatment can inhibit this activation. At the same time, M1 markers were lower, and M2 markers were obviously higher after combined AB and TSIIA treatment compared to treatment with AB alone. These results show that polarization from M1 to M2 may participate in the effect of TSIIA on myelin sheath protection and remyelination. Previous studies have shown that TSIIA neuroprotection was associated with less microglial activation and reduced expression of NADPH oxidase and iNOS 21, 24, 41. Our results further indicate that TSIIA suppresses M1 microglia activation and promotes M2 microglia activation. Others have shown that M2 cells drive OL differentiation during remyelination and that this is an essential part of an effective remyelination response (Miron and Franklin, 2014)^39^. Our study showed that TSIIA reduces demyelination and promotes remyelination by polarizing microglial cells from M1 to M2. In accordance with the abovementioned research of the effect of M2 cells on remyelination, it be the reason that AB combined with TSIIA is superior to AB alone to treat *A. cantonensis*-induced demyelination.

## Conclusion

Although previous studies have investigated multiple treatments that may attenuate demyelination and promote remyelination, this is the first evidence that TSIIA can alleviate demyelination caused by *A. cantonensis* and subsequently promote remyelination. Furthermore, our results suggest that polarization of microglia from M1 to M2 is one of the key mechanisms by which TSIIA affects demyelination and remyelination. TSIIA combined with AB may be an effective method to treat demyelination caused by *A. cantonensis* infection and other demyelinating diseases including MS.

## Figures and Tables

**Figure 1:**
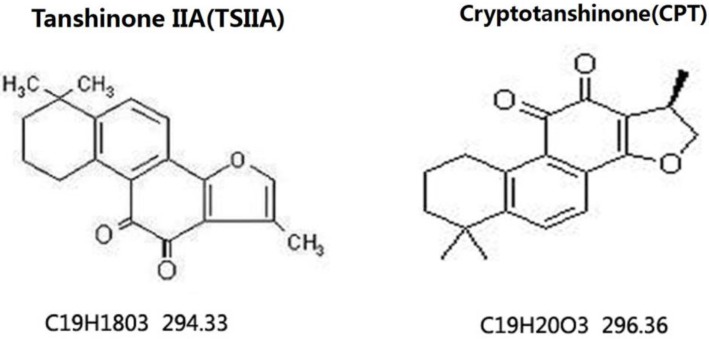
** Chemical structures of TSIIA and CPT 42.** TSIIA and CPT are both extracted from Danshen and have different chemical structures and molecular weights.

**Table 1:** Primer sequences for real-time PCR

Gene name	Primer sequence
iNOS	Forward 5'-ACCTTGTTCAGCTACGCCTT-3'
	Reverse 3'-CATTCCCAAATGTGCTTGTC-5'
IL-1β	Forward 5'-TGTGAAATGCCACCTTTTGA-3'
	Reverse 3'-TGTCCTCATCCTGGAAGGTC-5'
TNF-α	Forward 5'-GAACTGGCAGAAGAGGCACT-3'
	Reverse 3'-AGGGTCTGGGCCATAGAACT-5'
IL-6	Forward 5'-CTGATGCTGGTGACAACCAC-3'
	Reverse 3'-CAGAATTGCCATTGCACAAC-5'
β-actin	Forward 5'-GGCATCCTGACCCTGAAGTA-3'
	Reverse 3'-CTCTCAGCTGTGGTGGTGAA-5'
IL-5	Forward 5'-CACCAGCTATGCATTGGAGA-3'
	Reverse 3'-TTTGGCGGTCAATGTATTTCT-5'
IL-13	Forward 5'- AGCATGGTATGGAGTGTGGA-3'
	Reverse 3'-TTGCAATTGGAGATGTTGGT-5'
CD86	Forward 5'-GTCTAAGCAAGGTCACCCGAAAC-3'
	Reverse 3'-TCCAGAACACACACAACGGTCATA-5'
CD206	Forward 5'- GCATCAGTGAAGGTGGATAGAG-3'
	Reverse 3'-TGAGAACAACAGACAGGAGGAC-5'
CD11b	Forward 5'- CACGACGAGCACAAGTCCAAC-3'
	Reverse 3'-AGTCCTCAACTCCACGGGTCA-5'
Arg-1	Forward 5'- TGTCTGCTTTGCTGTGATGCC-3'
	Reverse 3'-GGAACTCAACGGGAGGGTAAC-5'
Ym-1	Forward 5'- GACGAAGGAATCTGATAACTGACTG-3'
	Reverse 3'-CATTGGAGGATGGAAGTTTGG-5'

## Abbreviations

TSIIA: tanshinone IIA
AB: albendazole
OL: oligodendrocyte
MBP: myelin basic protein
IL: interleukin
TNF: tumor necrosis factor
iNOS: inducible nitric oxide synthase
CNS: central nervous system
MS: multiple sclerosis
CPT: cryptotanshinone
PBS: phosphate-buffered saline
DMSO: dimethyl sulfoxide
HE: hematoxylin and eosin
LFB: luxol fast blue
TEM: transmission electron microscopy
BSA: bovine serum albumin
TRITC: tetramethylrhodamine
FITC: fluorescein isothiocyanate
BCA: bicinchoninic
